# The Seton in Chronic Patellar Bursitis

**Published:** 1880-02

**Authors:** 


### THE SETON IN CHRONIC PATELLAR BURSITIS.

In the *London Lancet* for December, Dr. Austin recommends the following as a certain method for the cure of chronic housemaid's knee: The seton, composed of a double silk thread, moistened in weak carbolic acid, is conveniently passed through the same canula which draws off the fluid, and in certain circumstances, when the avoidance of pain is urgently required, the ether spray will be found a great boon. Too small a trocar is not to be selected, or the apertures will be apt to close up and obstruct the discharges. To have to enlarge them afterwards would be exceedingly disagreeable. A pad of lint, moistened also with carbolic oil, covered with gutta-percha tissue, and the whole covered by a few turns of a bandage, is both agreeable to the patient, and helps to maintain the patency of the apertures. The seton should be drawn every morning, in order to present a fresh portion of it each time to the suppurating interior, and the pus encouraged to ooze out by frequent and gentle pressure of the fingers. In five or six days, the seton may be withdrawn, and after five or six days more of rest, the patient may be allowed to walk about. Should any congestion or weakness be left behind, it is effectually removed by the local use of the cold douche. Iodine, blistering, pressure, and even simple cupping, are very uncertain remedies.

